# Spent Mushroom Substrate Improves Microbial Quantities and Enzymatic Activity in Soils of Different Farming Systems

**DOI:** 10.3390/microorganisms12081521

**Published:** 2024-07-24

**Authors:** Maša Pintarič, Ana Štuhec, Eva Tratnik, Tomaž Langerholc

**Affiliations:** Department of Microbiology, Biochemistry, Molecular Biology and Biotechnology, Faculty of Agriculture and Life Sciences, University of Maribor, Pivola 10, 2311 Hoče, Slovenia; ana.stuhec1@student.um.si (A.Š.); eva.tratnik1@student.um.si (E.T.); tomaz.langerholc@um.si (T.L.)

**Keywords:** conventional–integrated, organic, biodynamic, soil microorganisms, soil enzymatic activity, spent mushroom substrate

## Abstract

Organic fertilizers, such as spent mushroom substrate (SMS), improve soil fertility, but studies comparing their effects on different agricultural soils are limited. In this study, the effects of standard, SMS and composed fertilizers on soils from conventional–integrated, organic and biodynamic farming were investigated. Soil samples were analyzed for microorganisms and the activity of β-glucosidase (β-GLU), β-1,4-N-acetylglucosaminidase (NAG), urease (URE), arylamidase (ARN), phosphatase (PHOS), acid phosphatase (PAC), alkaline phosphatase (PAH) and arylsulphatase (ARS). Biodynamic soil showed the highest microbial counts and enzyme activities, followed by organic and conventional soils. SMS significantly increased the number of microorganisms and enzyme activities, especially in biodynamic and organic soils. Seasonal variations affected all microorganisms and most enzymes in all soils, except NAG in conventional and organic soils. Biodynamic soil showed stable activity of enzymes and microorganisms throughout the year, indicating greater stability. This study concludes that soil microorganisms and enzyme activities respond differently to fertilization depending on the soil type, with SMS demonstrating beneficial effects in all tested soils.

## 1. Introduction

Climate, natural events, urbanization, agriculture and forestry, waste disposal, etc., are known stress factors that affect soil health, leading to potential soil degradation and impairing its original role in nature, i.e., its function as a vital system [1]. A primary goal in agriculture is to improve soil fertility in order to increase crop productivity by increasing nutrients in the soil [2]. Nutrient retention can be controlled through the use of various fertilizers, of which synthetic chemical fertilizers can have negative effects on the environment (including soil, air and water) and human and animal health if used excessively and inappropriately [3,4,5,6]. One of the alternative fertilizers that is organic in nature is spent mushroom substrate (SMS), a major by-product of the mushroom production industry. SMS is a source of mushroom mycelium residues, lignocellulosic biomass and additives (lime, gypsum dust) and contains a high proportion of organic matter and many enzymes [7]. The composition of SMS is highly variable, with the ratio of substances contained in SMS largely depending on the origin of the fungal culture medium [8,9]. The main weakness of this mushroom substrate is its stability, which, if not perfect, can limit its wide use in agriculture. Nevertheless, this problem can be solved by composting alone or in combination with other crop residues and under controlled conditions [10].

Microbiological activity in soil plays a key role in soil alongside other organisms and plants [11]. As microorganisms are known to respond rapidly to environmental stress and therefore adapt quickly to various changes, they are already known to be excellent indicators of soil health [12,13]. Soil biological activity is mainly concentrated in the upper layer, at a depth of several centimeters to 30 cm. In the upper part of the soil, the biological components, consisting mainly of microorganisms, occupy only a small part (<0.5%) of the total soil volume and account for less than 10% of the total organic matter in the soil, but they significantly influence biochemical processes that take place in the soil [14,15]. Microorganisms are involved in the transformation and degradation of synthetic organic compounds and produce compounds that affect the physical properties of the soil and its pH. In this way, they are involved in the dissolution and formation of minerals and also influence water retention, infiltration rate, crust formation and susceptibility to erosion [12,13,15,16,17]. They are also the main players in organic carbon and nutrient cycling [13] and interact closely with plants [18]. Soil microorganisms are extremely numerous and taxonomically diverse. It is estimated that billions of microbial cells and thousands of species are present in one gram of soil [12]. In general, bacteria (15% of the total living biomass) and fungi (2%) are the predominant microorganisms in soil with more biomass than protists and archaea (1%) [2,13,17]. The class Actinomycetia [19] (also known as actinomycetes) includes Gram-positive bacteria belonging to the phylum Actinomycetota (formerly Actinobacteria) [20]. These bacteria are found in terrestrial and aquatic habitats and are an important component of the bacterial community, especially under extreme environmental conditions such as high pH, high temperature or water stress [21]. Actinomycetes play a major role in maintaining soil ecology [22]. Fungi can adapt to different environmental conditions due to their high flexibility and efficiency. It is therefore not surprising that they can be found in almost all environments and can survive under variable temperatures and pH values [23,24]. However, the diversity and activity of fungi are controlled by various factors such as structures of soil or plant, temperature, water and pH [23]. 

Enzymes act as biological catalysts that accelerate biochemical reactions in living organisms [25]. Soil enzymes serve as biological indicators of soil quality and reflect soil biology. The extracellular enzymes are released into the environment by the action of microorganisms in the soil or by the root system of plants and then bind to particles in the soil or remain in free form [26]. To evaluate the factors controlling the decomposition of plant litter and soil quality, enzymes involved in the degradation of the main components of the waste material and hydrolases related to different nutrient cycles are most commonly used. In the context of the carbon cycle, the enzymes β-glucosidase (β-GLU), β-galactosidase, and β-1,4-N-acetylglucosaminidase (NAG) play crucial roles. In the nitrogen cycle, the enzymes urease (URE) and arylamidase (ARN) are significant, while phosphatase (PHOS) is utilized in the phosphorus cycle. Moreover, arylsulphatase (ARS) is employed in the sulfur cycle. Other enzymes in soil can be amylase, amidase, phenol oxidase, cellulase, chitinase, dehydrogenase and protease [27].

Soil microbiome structure and its enzymatic activity depend on several factors, including characteristics of the soil environment and characteristics of the soil microbiome itself. The latter is influenced by biotic factors, which include various bacteria, fungi, archaea, viruses, protozoa and plant species, as well as abiotic factors, which are of paramount importance and include soil structure and type, pH, nutrients, and geographic and climatic factors [2]. Considering all these different aspects, we aimed to evaluate and compare the effects of different fertilization types on the number of total microorganisms, actinomycetes and fungi, as well as on their enzymatic activity in three different agricultural soils in Slovenia (from conventional–integrated, organic and biodynamic farming). The integrated production is a more environmentally friendly production method. By using natural resources and mechanisms that reduce the negative impact of agriculture on the environment and human health, high-quality agricultural products are produced. In Slovenia, integrated cultivation of crops, fruit, grapes and vegetables is practiced. The production technology, control procedures and type of labeling are defined in the regulations on integrated production and the technological instructions for integrated production [28], which are issued annually by the Ministry of Agriculture, Forestry and Food [29]. Organic farming in Slovenia follows the guidelines of Regulation (EU) 2018/848 of the European Parliament and of the Council on organic production and labeling of organic products [30]. Biodynamic agriculture in Slovenia follows the guidelines and standards of the agriculture association The Biodynamic Federation Demeter International [31].

## 2. Materials and Methods

### 2.1. Location and Experimental Design

This study took place from 2022 to 2023 on three different farms in north-eastern Slovenia, Europe. The exact location sites and characteristics of the three farms are summarized in Table 1. More detailed compositions of the applied fertilizers can be found in Supplementary Tables S1 and S2.

The experimental plots were randomly laid out in triplicate (the area of a single plot was 7.56 m^2^, 5.88 m^2^ and 2.94 m^2^ for farms M, S and T, respectively), and fertilization was applied over two years in springtime according to the experimental design:

- Soil without fertilizer (C: control);
- Soil with single dose of composted spent mushroom substrate (SMS);
- Soil with single dose of standard fertilization (SF);
- Soil with half dose of SF and half dose of SMS (SF.SMS).

The composition of the solid mushroom substrate was based on straw (wheat, barley and rye), calcium sulfate (Ca_2_SO_4_) and calcium carbonate (CaCO_3_). It did not contain any mineral additives, as it was solely intended for organic farming. It had undergone two mushroom production cycles (*Pleurotus*). The SMS was thoroughly composted aerobically before use. The amount of SMS added was introduced as an equivalent, calculated on the added nitrogen compared to the SF treatment. Two tomato varieties were planted as test plants in farms M, S and T (“Belle” and “San Marzano”, respectively) in plastic greenhouses with PVC soil cover (farm M and S) and green mulch cover (farm T). All farms have set up an irrigation system. The characteristics of the initial soil and the SMS on the three farms are shown in Table 2.

### 2.2. Soil Sampling

Two years after the start of the project (2023), soil samples were taken for the analysis of microorganisms and enzymes. Sampling was performed in each season (winter (January), spring (April), summer (July) and autumn (October)) for microbial population and enzyme analysis. Then, 500–1000 g of representative samples within each tested plot were collected at 20 randomly selected locations from a layer of 0–10 cm using a soil sampler. The soil sampler was cleaned with distilled water and disinfected with 70% ethanol before each use. When sampling, the outermost edges of the plots and the immediate vicinity of the root system of the planted plants were avoided. Samples were carefully sieved through a 2 mm mesh sieve, transferred into zippered polyethylene bags (220 × 250 mm) that were previously sterilized under UV light (overnight) and transported to the laboratory in a cooler bag with ice packs. In the laboratory, the samples were thoroughly mixed, homogenized and stored at 4 °C. Analysis was performed within 24 h and 4 days (for microbiological analysis and enzyme analysis, respectively).

### 2.3. Processing Soil Samples for Soil Moisture

To calculate the gravimetric soil moisture [32], 50.0 g of mixed soil samples was weighed into numbered glass Petri dishes of known mass and dried in a preheated dryer at 104 °C for 24 h. After 24 h, the Petri dishes with the samples were placed in a desiccator for 30–45 min to cool, and then weighed again. The gravimetric moisture content (%) of the soil was calculated using Equation (1):

(1) $$\frac{\;m-d}{d}\times 100,$$

where ‘*m*’ is the mass of the wet sample (g) and ‘*d*’ is the mass of the dry sample (g).

### 2.4. Processing Soil Samples for Microbial Population Analysis 

A total of 10.0 g of the sample was weighed and transferred to sterile microperforated 400 mL filter bags (BagPage^®^, Interscience, Saint Nom la Brétèche, France). Subsequently, 90 mL of buffered peptone water (Millipore, Merck, Darmstadt, Germany) was added and homogenized in a laboratory blender BagMixer 400 SW (Interscience, Saint Nom la Brétèche, France) for 2 min. Quantitative analysis of the microbial population was performed using the standard serial dilution method, followed by the spreading technique on agar plates. Tryptic Soy Agar, Actinomycete Isolation Agar and Potato Dextrose Agar (all agars are from Millipore, Merck, Darmstadt, Germany) were used for isolation and enumeration of the total number of microorganisms, actinomycetes and fungi (yeasts and molds), respectively. The plates were incubated according to the manufacturer’s instructions. The results were analyzed after colony counting and calculated using the following Equation (2):

(2) $$N=\frac{\varepsilon C}{V\times \left(n1+0.1\times n2\right)\times d},$$

where *N* is the number of microorganisms present in the test sample (expressed as colony-forming units per g of wet sample [CFU/g of wet soil]; *C* is the sum of the colonies counted on the two dishes retained from two successive dilutions; *V* is the volume of sample inoculum, added on each agar plate [mL]; *n*1 is the number of agar plates corresponding to the first retained dilution; *n*2 is the number of agar plates corresponding to the second retained dilution; and *d* is the dilution corresponding to the first dilution retained. Finally, the results were expressed as the number of colonies per g of dry sample [CFU/g of dry sample], taking into account the gravimetric moisture content.

### 2.5. Processing Soil Samples for Enzyme Analysis

The samples for enzyme analysis were processed according to the instructions of the standard ISO 20130:2018 [33] with slight differences. Ten grams of the sample was weighed and transferred into sterile microperforated 400 mL filter bags (BagPage^®^, Interscience, Saint Nom la Brétèche, France). A suitable buffer (1:4) was then added to the sterile bags containing the samples and homogenized for 2 min in a BagMixer 400 SW laboratory mixer (Interscience, Saint Nom la Brétèche, France). Preparation of buffers and reagents, sample plates, addition of substrates and absorbance measurements were performed according to the instructions of the ISO 20130:2018 standard. Standard curves for paranitrophenol (PNP; λ = 405 nm), ammonium chloride (NH_4_Cl; λ = 650 nm) and β-naphthylamine (λ = 540 nm) were plotted and the result was calculated using the following Equation (3):

(3) $$A=\frac{\left(Cs-Cb\right)\times D\times Vss}{RT\times Wds},$$

where *A* is the enzymatic activity in [mU/g] of dry sample [nmol/min/g of dry sample]; Cs is the concentration of product formed in the sample [nmol/mL]; *Cb* is the concentration of product formed in blank [nmol/mL]; *D* is the dilution of the sample in microplates; *Vss* is the volume of the sample solution [mL]; *RT* is the reaction time [min]; and *Wds* is the mass of dry sample [g]. The enzyme activity measured in this study was arylamidase (ARN; E.C. 3.4.11.2), arylsulfatase (ARS; E.C. 3.1.6.1), β-glucosidase (β-GLU; E.C. 3.2.1.21), N-acetyl-glucosaminidase (NAG; E.C. 3.2.1.52), phosphatase (PHOS; E.C.3.1.4.1), acid phosphatase (PAC; E.C.3.1.4.1), alkaline phosphatase (PAK; E.C.3.1.4.1) and urease (URE; E.C. 3.5.1.5).

### 2.6. Statistical Analysis

The data obtained from the microbial and enzymatic analysis were analyzed using SPSS IBM Statistics 24.0 (IBM Inc., Armonk, NY, USA). First, the data were analyzed using the Shapiro–Wilk normality test. Subsequently, analyses were performed using ANOVA, followed by a post hoc analysis using the Bonferroni test or the Dunnett T3 test after the Levene test for homogeneity of variances. The data that did not meet the assumptions were analyzed with the Kruskal–Wallis H-test. The α-level was set at 5%, and a *p*-value < 0.05 was considered statistically significant.

## 3. Results

### 3.1. Effect of Different Fertilization Types and Seasonal Changes in Three Farms

#### 3.1.1. Microorganism Quantities

Quantification of microorganisms was based on the count of actinomycetes, the total number of microorganisms and fungi.

- Farm M

**Figure 1:**
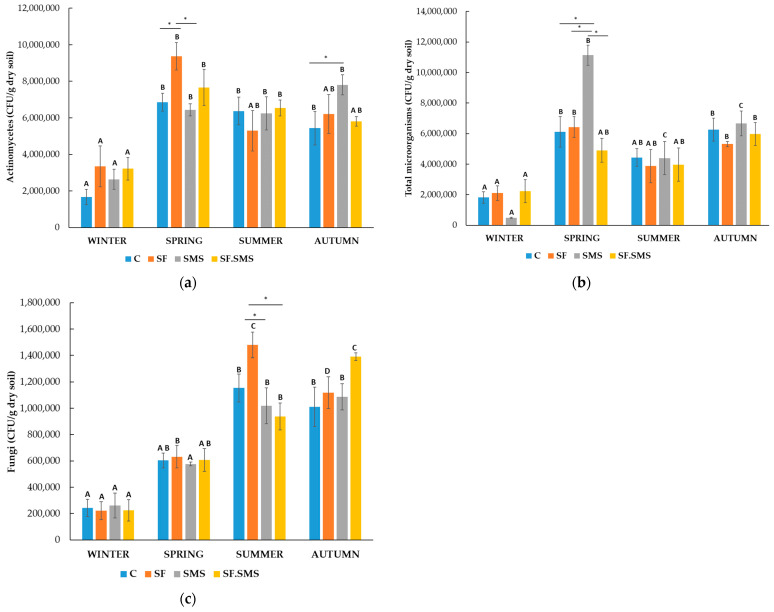
Effect of different fertilization types and seasonal changes in microorganism quantities in farm M. Number of actinomycetes (**a**); number of total microorganisms (**b**); number of fungi (**c**). Different uppercase letters indicate significant differences between the seasons in the same fertilizer treatment (*p* < 0.05). Asterisk (*) indicates a significant difference between different fertilizers in the same season (*p* < 0.05). Data from three independent experiments are presented as medians (geometrical shapes) with ranges. Soil without fertilizer (C: C group); soil with single dose of composted spent mushroom substrate (SMS); soil with single dose of standard fertilization (SF); soil with half dose of SF and half dose of SMS (SF.SMS).

The quantities of actinomycetes are shown in Figure 1a. Differences between the different fertilizer types and the controls were observed in each season. In winter and autumn, all fertilization types increased the number of actinomycetes, while in spring and summer, the effect of fertilization varied. In autumn, the number of actinomycetes increased after all fertilization types, but a statistically significant difference was found only between the SMS and the C group (*p* = 0.035). In addition, SF in spring significantly increased the amount of actinomycetes compared to the C group (*p* = 0.012) and the SMS group (*p* = 0.005). However, the differences in winter and summer were not statistically significant. Furthermore, as expected, the amount of actinomycetes was lowest in winter (1.7 × 10^6^–3.3 × 10^6^ CFU/g dry soil) in all the groups compared to other seasons. In the C group and the SMS group, the amount of actinomycetes increased significantly in spring (*p* = 0.005 and *p* = 0.007, respectively), summer (*p* = 0.013 and *p* = 0.012, respectively) and autumn (*p* = 0.027 and *p* < 0.001, respectively) compared to the amount in winter. In the SF group, a statistically significant difference in the amount of actinomycetes was only observed between spring and winter (*p* = 0.010). Finally, in the SF.SMS combination group, the amount of Actinomycetes was statistically significantly increased in spring (*p* = 0.024) and summer (*p* = 0.047) compared to the amount in winter. A very high number of total microorganisms was observed in the SMS group in spring (Figure 1b). In fact, there was a statistically significant difference between the SMS and the other three groups (C group: *p* = 0.002, SF: *p* = 0.003 and SF.SMS: *p* < 0.001). In winter, a significant increase in the total number of microorganisms was also observed in the SF and SF.SMS groups compared to the SMS group (*p* = 0.031 and *p* = 0.024, respectively). In the other seasons, the addition of different fertilizers caused only small differences, none of which were significant. In addition, seasonal variations in the total number of microorganisms were observed. As with the actinomycetes (Figure 1a), the lowest number of total microorganisms was found in winter (0.5 × 10^6^–2.2 × 10^6^ CFU/g dry soil). In the C and SF groups, the number of total microorganisms increased significantly in spring (*p* = 0.005 and *p* < 0.001, respectively) and autumn (*p* = 0.004 and *p* = 0.003, respectively) compared to the amount in winter. In the SF.SMS group, a statistically significant difference in the total amount of microorganisms was only observed between autumn and winter (*p* = 0.044). In addition, the greatest variation in the total amount of microorganisms between seasons was observed in the SMS group. The number of total microorganisms increased significantly in spring, summer and autumn (all *p* < 0.001) compared to the number in winter. In addition, the number of microorganisms was also statistically different between spring and summer/autumn (both *p* < 0.001). The number of fungi in all groups within each season is shown in Figure 1c. There was a small difference in the number of fungi between the groups in winter and spring. Furthermore, SF in summer seems to have the greatest effect on the number of fungi compared to the other groups. In fact, a statistically significant increase was found compared to the SMS and SF.SMS groups (*p* = 0.019 and *p* = 0.011, respectively), but not to the C group. In the autumn, the SF.SMS group had the largest number of fungi compared to the other groups, although not significantly. Overall, the number of fungi in the fertilization groups (SF, SMS and SF.SMS) seems to be most influenced by the seasonal changes. Indeed, as expected, the number of fungi in all groups was lowest in winter (2.2 × 10^5^–2.6 × 10^5^ CFU/g dry soil) compared to the other seasons. In the SF group, the number of fungi is significantly higher in spring, summer and autumn than in winter (all *p* < 0.001). In addition, a statistically significant difference in the number of fungi between all seasons was also found within this group. A statistically significant increase in the number of fungi was found in summer and autumn compared to spring (*p* < 0.001 and *p* = 0.004, respectively). Finally, a statistically significant difference in the number of fungi was found between summer and autumn (*p* < 0.001). Seasonal changes also affected the number of fungi in the SMS and SF.SMS groups. In fact, a significant increase was observed in the SMS group in summer and autumn compared to winter (both *p* < 0.001) and compared to spring (*p* = 0.010 and *p* = 0.004, respectively). A similar pattern was observed in the SF.SMS group. There was a significant increase in fungi quantities in summer and autumn compared to winter (*p* = 0.004 and *p* < 0.001, respectively) and a significant increase in autumn compared to spring (*p* = 0.001) and summer (*p* = 0.026). The seasonal changes had a significant effect at least on the number of fungi in the C group. A statistically significant difference was only found between winter and summer/autumn (*p* = 0.003/*p* = 0.005).

- Farm T

**Figure 2:**
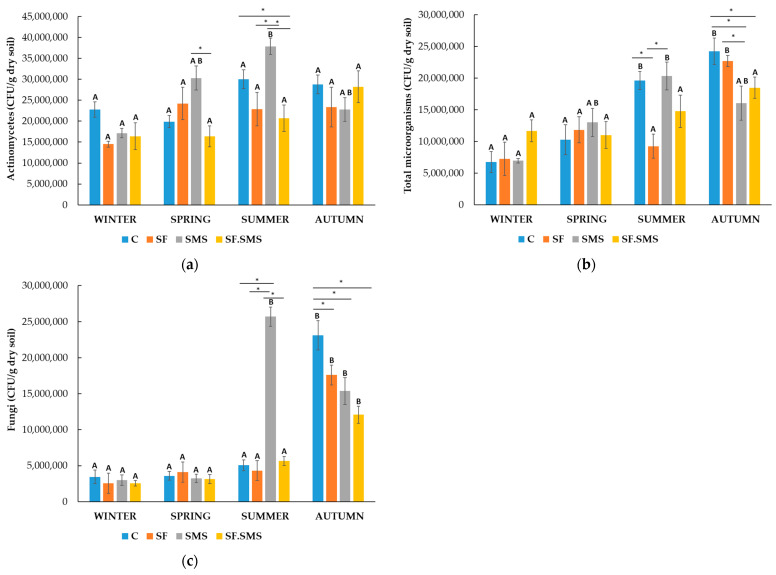
Effect of different fertilization types and seasonal changes in microorganism quantities in farm T. Number of actinomycetes (**a**); number of total microorganisms (**b**); number of fungi (**c**). Different uppercase letters indicate significant differences between the seasons in the same fertilizer treatment (*p* < 0.05). Asterisk (*) indicates a significant difference between different fertilizers in the same season (*p* < 0.05). Data from three independent experiments are presented as medians (geometrical shapes) with ranges. Soil without fertilizer (C: control); soil with single dose of composted spent mushroom substrate (SMS); soil with single dose of standard fertilization (SF); soil with half dose of SF and half dose of SMS (SF.SMS).

Compared to farm M (Figure 1), the number of all microorganisms detected in farm T (Figure 2) was at least 5 to 10 times higher. There was no statistical difference in the amount of actinomycetes (Figure 2a) between the winter and autumn groups. However, a statistical difference in the number of actinomycetes was found between the SMS and SF.SMS groups in spring (*p* = 0.008) and summer, with the SMS fertilization showing a statistically significant difference from the SF (*p* = 0.005) and SF.SMS (*p* < 0.001) groups, but not from the C group. The number of actinomycetes increased significantly only in the C group compared to the SF.SMS group (*p* = 0.032). In summer and spring, however, the SMS fertilization had the greatest effect (3.8 × 10^7^ CFU/g dry soil and 3.0 × 10^7^ CFU/g dry soil, respectively), compared to the other types of fertilization, followed by winter. Interestingly, statistical differences due to seasonal dynamics were only observed in the SMS group. Indeed, the number of actinomycetes was significantly higher only in summer compared to winter (*p* < 0.035). Figure 2b shows the number or total of microorganisms determined in farm T. The effect of the different fertilizations could be observed in all seasons, although statistically significant differences were only found between the groups in summer and autumn. In summer, the total number of microorganisms was significantly higher in the C group (*p* = 0.002) and in the SMS group (*p* = 0.001) than in the SF group. In autumn, the total number of microorganisms was significantly higher in the C group than in the SMS group (*p* = 0.006) and in the SF.SMS group (*p* = 0.040). In addition, SMS fertilization significantly reduced the total number of microorganisms compared to SF (*p* = 0.021). Seasonal changes had a significant effect on the total number of microorganisms in the C group, the SF group and the SMS group, but not in the SF.SMS group. In the C group, a statistically significant difference was observed between winter and summer/autumn (both *p* < 0.001). There was also a significant difference in the number between summer and spring (*p* = 0.002). In the SF group in autumn, the total number of microorganisms was significantly higher than in summer (*p* = 0.008), spring (*p* = 0.028) and winter (*p* = 0.004). The highest number of fungi was found in the SMS group (2.6 × 10^7^ CFU/g dry soil) in summer (Figure 2c), resulting in a statistically significant difference compared to the C group, the SF and the SF.SMS group (*p* < 0.001) in the same season. However, such a drastic effect of SMS fertilization was not observed in other seasons. In autumn, the C group had the highest statistically significant number of fungi (2.3 × 10^7^ CFU/g dry soil) compared to the other groups (SF: *p* = 0.021, SMS: *p* = 0.003, SF.SMS: *p* < 0.001). Summer and autumn were the only seasons that affected the number of fungi within the groups. In fact, the number of fungi was significantly higher in fall than in spring and winter for the C group, the SF group and the SF.SMS group (all *p* < 0.001). In the SMS group, the fungi number was significantly reduced in winter compared to summer (*p* < 0.001) and autumn (*p* = 0.008) and in spring compared to summer (*p* < 0.001) and autumn (*p* = 0.009).

- Farm S

**Figure 3:**
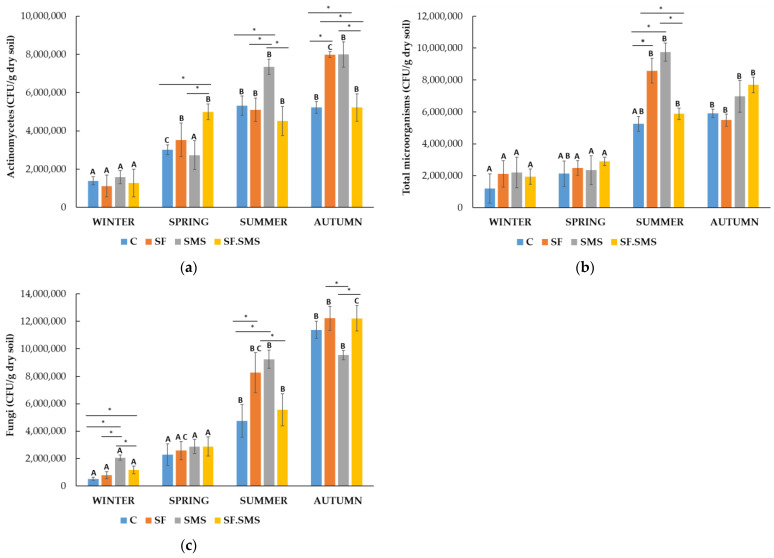
Effect of different fertilization types and seasonal changes in microorganism quantities in farm S. Number of actinomycetes (**a**); number of total microorganisms (**b**); number of fungi (**c**). Different uppercase letters indicate significant differences between the seasons in the same fertilizer treatment (*p* < 0.05). Asterisk (*) indicates a significant difference between different fertilizers in the same season (*p* < 0.05). Data from three independent experiments are presented as medians (geometrical shapes) with ranges. Soil without fertilizer (C: control); soil with single dose of composted spent mushroom substrate (SMS); soil with single dose of standard fertilization (SF); soil with half dose of SF and half dose of SMS (SF.SMS).

All fertilization types affected the number of actinomycetes (Figure 3a), compared to the C groups in all seasons. The addition of SMS fertilizer increased the number of actinomycetes in all seasons but spring. In fact, a significant overall increase in SMS was observed compared to the C group (*p* = 0.017), the SF group (*p* = 0.010) and the SF.SMS group (*p* = 0.002). However, the lowest non-significant effect of all fertilizers on the number of actinomycetes was observed in winter (1.1 × 10^7^ CFU/g dry soil–1.6 × 10^7^ CFU/g dry soil) and the greatest in autumn (4.5 × 10^7^ CFU/g dry soil–8.5 × 10^7^ CFU/g dry soil). In fact, the SF and SMS groups showed a significant increase in actinomyces in autumn (both *p* < 0.001) compared to the C group. Moreover, the SF.SMS group showed a significant reduction in actinomycetes compared to the SF and SMS groups (both *p* = 0.001). Finally, in spring, the SF.SMS group had the highest number of actinomycetes (4.9 × 10^7^ CFU/g dry soil) compared to the C group (*p* = 0.029) and the SMS group (*p* = 0.016), which was statistically significant. The seasonal changes showed even more significant differences within individual groups. In the C group, the number of actinomycetes increased in spring (*p* = 0.002), summer (*p* < 0.001) and autumn (*p* < 0.001) compared to winter. There was also a statistically significant difference in the number of actinomycetes between spring and autumn (*p* < 0.001) and spring and summer (*p* < 0.001) in the same group. The number of actinomycetes in the SF group was significantly increased in spring (*p* = 0.008), summer (*p* < 0.001) and autumn (*p* < 0.001) compared to winter. Statistically significant differences were also observed between autumn and spring/summer (*p* < 0.001/*p* = 0.003). Some significant seasonal differences were also observed in the SMS group. Here, the number of actinomycetes increased significantly in summer (*p* = 0.013) and autumn (*p* = 0.004) compared to winter. In addition, a statistically significant difference in the number of actinomycetes was also found between spring and autumn (*p* = 0.014). In the combined group (SF.SMS), the number of actinomycetes was significantly increased in spring (*p* = 0.001), summer (*p* = 0.002) and autumn (*p* = 0.001) compared to winter. The highest increase in the total number of microorganisms (Figure 3b) after each type of fertilization was observed in summer (5.8 × 10^6^ CFU/g dry soil–9.7 × 10^6^ CFU/g dry soil), followed by the one in autumn (7.0 × 10^6^ CFU/g dry soil–7.6 × 10^6^ CFU/g dry soil) (except for the SF group). A statistically significant difference in the total number of microorganisms between the C group and the SF/SMS group was also observed in summer (both *p* < 0.001). In addition, the SF/SMS group significantly reduced the number of total microorganisms compared to the SF (*p* = 0.002) and SMS (*p* < 0.001) groups. In all other seasons, however, no significant differences were found between the groups. In winter and spring, the total microbial number was lowest in all groups (1.1 × 10^6^ CFU/g dry soil–2.2 × 10^6^ CFU/g dry soil and 2.1 × 10^6^ CFU/g dry soil–2.8 × 10^6^ CFU/g dry soil, respectively), followed by autumn and summer (5.4 × 10^6^ CFU/g dry soil–7.6 × 10^6^ CFU/g dry soil and 5.2 × 10^6^ CFU/g dry soil–9.7 × 10^6^ CFU/g dry soil, respectively). In fact, there was a statistically significant difference in the number of total microorganisms in the SF, SMS and SF.SMS groups between winter and summer/autumn (*p* = 0.005/*p* = 0.031, *p* < 0.001/*p* = 0.006 and *p* = 0.001/*p* = 0.004, respectively), followed by spring and summer/autumn (*p* = 0.005/*p* = 0.008, *p* < 0.001/*p* = 0.032 and *p* = 0.004/*p* = 0.017, respectively), but not in the C group. In the latest, a significant difference in the number of total microorganisms was found only between winter and autumn (*p* = 0.039). A few significant differences between the groups were also found after evaluating the number of fungi (Figure 3c). Although winter is the season with the lowest number of fungi (1.1 × 10^6^ CFU/g dry soil–8.0 × 10^6^ CFU/g dry soil), few significant differences were found between the groups in this season compared to the other seasons. In the SMS group, the number of fungi increased significantly compared to all three other groups (C group, SF: both *p* < 0.001 and SF.SMS: *p* = 0.007). The number of fungi also increased significantly in the SF.SMS group compared to the C group (*p* = 0.035). In summer, SMS fertilization again had a significant effect when compared with the C group (*p* = 0.009) and the SF.SMS group (*p* = 0.031). In the same season, a significant difference was found between the C group and the SF group after SF (*p* = 0.035). Finally, in autumn, the number of fungi in the SMS group was significantly lower than in the SF and SF.SMS groups (both *p* = 0.011). Seasonal fluctuations had a fairly large influence on the number of fungi in all groups. In the C group, the number of fungi was significantly lower in winter than in summer (*p* = 0.003) and fall (*p* < 0.001), but significantly higher in autumn than in spring and summer (both *p* < 0.001). A significant decrease in the number of fungi in winter compared to summer (*p* = 0.048) and autumn (*p* = 0.005) was also observed in the SF group. In addition, a statistically significant difference in fungi number was observed between spring and autumn (*p* = 0.013). A similar pattern as in the C group was also observed in the SMS group. The number of fungi was significantly lower in winter than in summer (*p* = 0.009) and autumn (*p* = 0.010), but significantly higher in summer (*p* = 0.017) and autumn (*p* = 0.019) than in spring. The SF.SMS group was most affected by the fluctuations in temperatures. In fact, the number of fungi increased significantly in summer (*p* = 0.048) and autumn (*p* < 0.001) compared to spring. The number of fungi was also significantly higher in autumn than in winter and summer (both *p* < 0.001). There was a significant increase in fungi number in summer as well, when compared to that in winter (*p* = 0.004). 

#### 3.1.2. Enzymatic Activity

- Farm M

**Figure 4:**
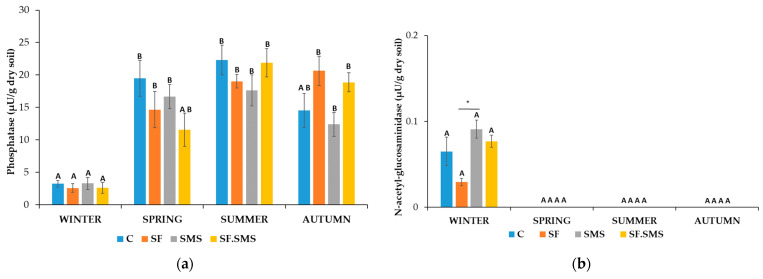

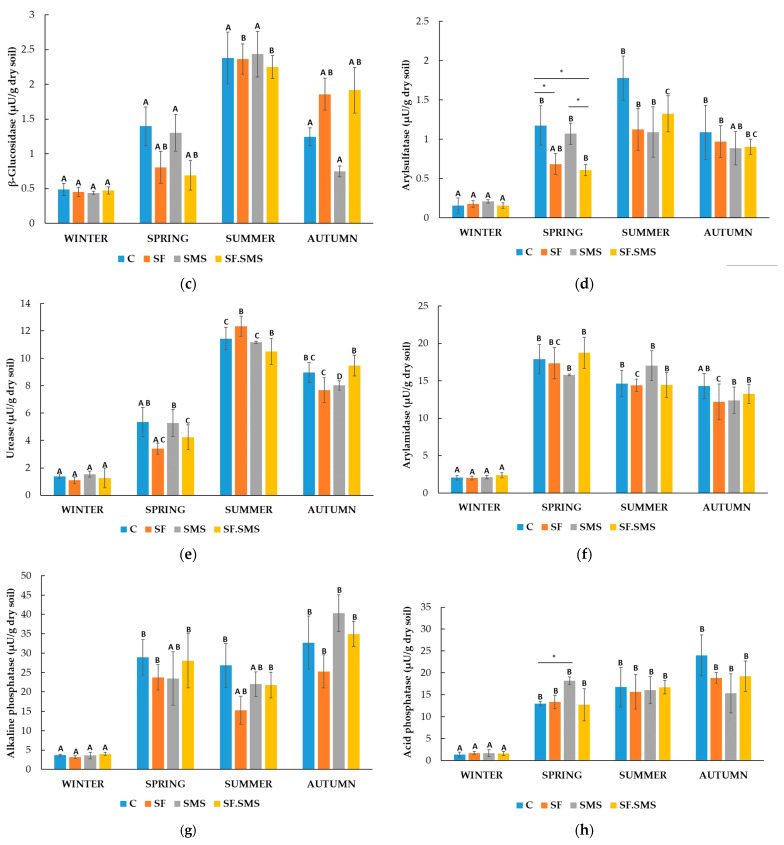
Effect of different fertilization types and seasonal changes in enzymatic activity in farm M. Enzymatic activity of phosphatase (**a**); enzymatic activity of N-acetyl-glucosaminidase (**b**); enzymatic activity of β-glucosidase (**c**); enzymatic activity of arylsulfatase (**d**); enzymatic activity of urease (**e**); enzymatic activity of arylamidase (**f**); enzymatic activity of alkaline phosphatase (**g**); enzymatic activity of acid phosphatase (**h**). Different uppercase letters indicate significant differences between the seasons in the same fertilizer treatment (*p* < 0.05). Asterisk (*) indicates a significant difference between different fertilizers in the same season (*p* < 0.05). Data from three independent experiments are presented as medians (geometrical shapes) with ranges. Soil without fertilizer (C: control); soil with single dose of composted spent mushroom substrate (SMS); soil with single dose of standard fertilization (SF); soil with half dose of SF and half dose of SMS (SF.SMS).

Figure 4a shows a reduced activity of phosphatase (PHOS) in all fertilization treatments compared to the control in all seasons except autumn. In autumn, PHOS activity even increased after SF and SF.SMS fertilization. However, differences in activity between the samples in all four seasons showed no statistical significance. Furthermore, seasonal dynamics were observed within each sample. In the C group, the activity of PHOS increased significantly in spring (*p* = 0.024) and summer (*p* = 0.026) compared to the activity in winter. A similar pattern was observed in the SF.SMS group, where enzyme activity was significantly increased in summer (*p* = 0.007) and autumn (*p* = 0.031) compared to winter. In the SF and SMS groups, a statistically significant increase in PHOS activity after winter was observed in spring (*p* = 0.009 and *p* < 0.001, respectively), summer (*p* = 0.001 and *p* < 0.001, respectively) and autumn (*p* = 0.003 and *p* < 0.014, respectively). Surprisingly, NAG activity was low and detected only in winter samples (Figure 4b). A statistically significant difference was observed between SF and SMS (*p* = 0.043). Despite not being statistically significant, SMS fertilization increased NAG activity at most (0.09 µU/g dry soil) compared to C. Nevertheless, the increased enzyme activity in winter showed no statistically significant differences compared to the other three seasons. Figure 4c shows no statistically significant differences between samples in each season for β-GLU. However, in spring and autumn, β-GLU activity was most strongly influenced by fertilization. Regardless of fertilization, activity was lowest in winter (0.44 µU/g dry soil) and highest in summer (2.43 µU/g dry soil). Statistically significant seasonal changes within the same samples were only observed in the SF and SF.SMS groups, where β-GLU activity increased in summer compared to winter (*p* = 0.007 and *p* < 0.035, respectively). A statistically significant effect of the different fertilizations compared to the C group was observed in arylsulfatase (ARS) activity in spring (Figure 4d). All types of fertilization reduced the activity of ARS. In the SF and SF.SMS groups, the activity was significantly reduced compared to the C group (both *p* = 0.005). In addition, ARS activity was significantly increased after SMS fertilization (*p* = 0.005) compared to the SF.SMS fertilization group. All types of fertilization also decreased the activity of ARS in summer and autumn, although the differences between samples within seasons were not statistically significant. Finally, the activity of the enzyme was lowest in winter (0.16–0.20 µU/g dry soil) compared to the other seasons in all samples. In fact, ARS activity was significantly increased in spring, summer and autumn in the C group (all *p* < 0.001) and the SF.SMS group (*p* = 0.018, *p* < 0.001 and *p* < 0.001, respectively). Compared to winter, ARS activity increased significantly in summer and autumn (*p* < 0.001 and *p* = 0.014, respectively) in the SF group and in spring and summer (*p* = 0.029 and *p* = 0.021, respectively) in the SMS group. In the SF.SMS group, ARS activity was significantly higher in summer compared to spring (*p* = 0.023). Figure 4e shows the dynamics of URE activity. In all seasons, except for winter, a small effect of different fertilization was evident. However, none of these effects were statistically different compared to the others. On the other hand, seasonal changes significantly affected the URE activity. URE activity significantly increased in spring, summer and autumn, compared to winter in the C (*p* = 0.005, *p* < 0.001 and *p* = 0.001, respectively), SMS (all *p* < 0.001) and SF.SMS (*p* = 0.016, *p* < 0.001 and *p* < 0.001, respectively) groups. URE activity in the SF group was significantly increased in summer and autumn (*p* < 0.001 and *p* = 0.005, respectively) compared to winter, and in summer compared to autumn (*p* = 0.038) and spring (*p* < 0.001). In the SMS group, there was also a statistically significant difference between the most enzymatically active seasons, i.e., between spring and summer/autumn (*p* < 0.001/*p* = 0.002) and summer and autumn (*p* = 0.045). As in the SMS group, URE activity in the SF.SMS group was significantly higher in summer and autumn than in spring (both *p* < 0.001). The effect of different fertilization types on ARN activity (Figure 4f) in each season was not statistically significant, although some increases and decreases were observed in the most active seasons (spring, summer and autumn). As expected, ARN activity in winter was significantly decreased compared to activity in spring/summer/autumn in the SF (all *p* < 0.001), SMS (*p* < 0.001/*p* = 0.05/*p* = 0.005) and SF.SMS groups (all *p* < 0.001). In the C group, ARN activity was significantly increased in spring and summer (*p* = 0.013 and *p* = 0.017, respectively) compared to activity in winter. However, a statistically significant difference between spring and autumn (*p* = 0.032) and between winter and autumn (*p* < 0.001) was also observed in the SF group. Figure 4g shows the activity of PAK. As we have already seen, the activity of this enzyme was not significantly affected by the different fertilization types in the individual seasons. Nevertheless, PAK activity was decreased in almost all groups compared to the C group in every season except for autumn. Regarding seasonal changes, PAK was most active in the fall compared to the other seasons for all groups except the SF group. In addition, PAK was least active in winter (3.18–4.01 µU/g dry soil) and the increase in its activity in spring, summer and autumn was statistically significant in the C group (*p* = 0.008, *p* = 0.009 and *p* = 0.003, respectively) and the SF.SMS group (*p* = 0.004, *p* = 0.019 and *p* < 0.001, respectively). In the SF group, however, the activity of PAK differed significantly between spring and winter (*p* = 0.023) and autumn and winter (*p* = 0.035). The latter pattern was also observed in the SMS group (*p* = 0.027). The PAC activity between the different groups was statistically significant only in spring (Figure 4h). In fact, PAC activity increased significantly in the SMS group compared to the C group (*p* = 0.034). The activity between the groups due to fertilization in the other seasons was not statistically different, although a decrease was observed in all groups compared to the C group in summer and autumn. When looking at seasonal changes, PAC was most active in the autumn compared to the other seasons in all groups except SMS. Winter was again the least enzymatically active season for all groups with statistically significant differences compared to spring, summer and autumn: C group (*p* = 0.045, *p* = 0.038 and *p* = 0.003, respectively), SF (*p* = 0.001, *p* < 0.001 and *p* < 0.001, respectively), SMS (*p* = 0.003, *p* = 0.006 and *p* = 0.009, respectively) and SF.SMS (*p* = 0.005, *p* < 0.001 and *p* < 0.001, respectively).

- Farm T

**Figure 5:**
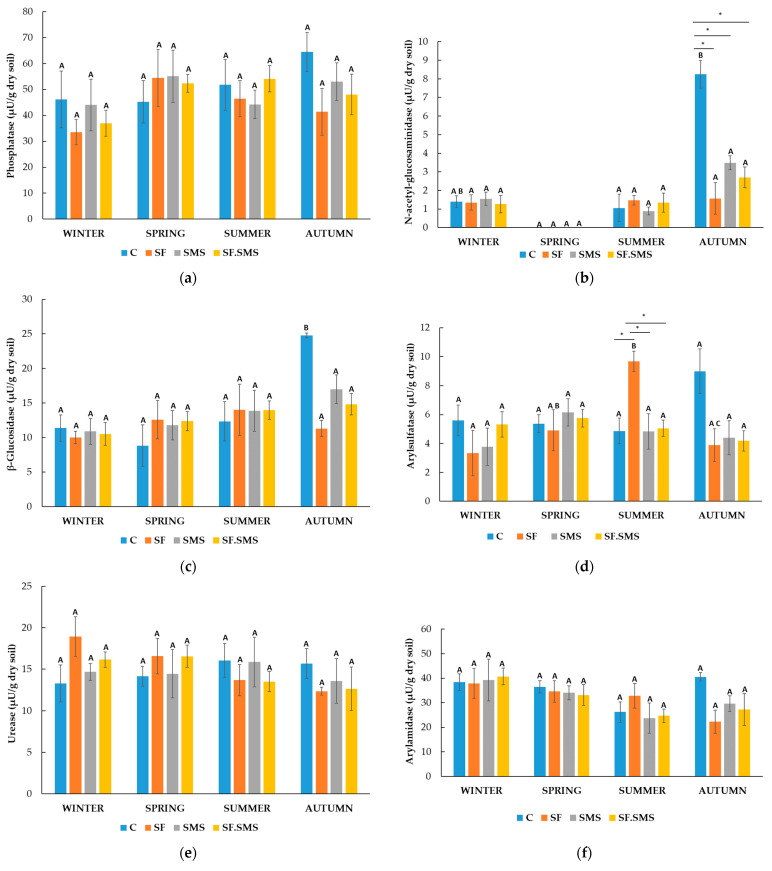

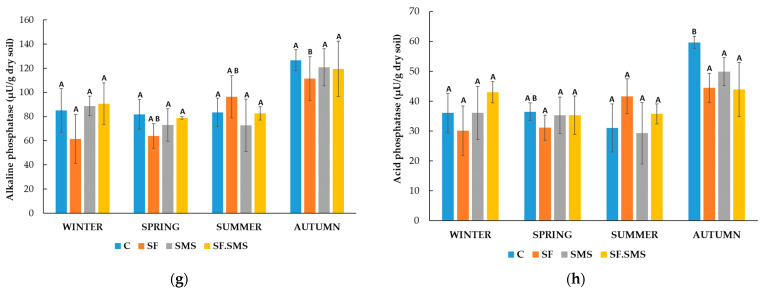
Effect of different fertilization types and seasonal changes in enzymatic activity in farm T. Enzymatic activity of phosphatase (**a**); enzymatic activity of N-acetyl-glucosaminidase (**b**); enzymatic activity of β-glucosidase (**c**); enzymatic activity of arylsulfatase (**d**); enzymatic activity of urease (**e**); enzymatic activity of arylamidase (**f**); enzymatic activity of alkaline phosphatase (**g**); enzymatic activity of acid phosphatase (**h**). Different uppercase letters indicate significant differences between the seasons in the same fertilizer treatment (*p* < 0.05). Asterisk (*) indicates a significant difference between different fertilizers in the same season (*p* < 0.05). Data from three independent experiments are presented as medians (geometrical shapes) with ranges. Soil without fertilizer (C: C group); soil with single dose of composted spent mushroom substrate (SMS); soil with single dose of standard fertilization (SF); soil with half dose of SF and half dose of SMS (SF.SMS).

A small effect of the different fertilization types on the activity of PHOS is shown in Figure 5a. The strongest decrease in activity compared to the C group was observed in autumn, but none of the differences between the groups were significant in any season. Furthermore, the seasonal changes did not statistically affect PHOS activity in any group. The highest activity of NAG was observed in autumn compared to the other seasons (Figure 5b). Interestingly, NAG activity was significantly reduced in the same season after all fertilization types compared to the C group (SF: *p* = 0.005; SMS: *p* = 0.038; SF.SMS: *p* = 0.024). There were no statistically significant differences in the activity of NAG between the groups in other seasons. Changes in the seasons affected only the C group. NAG activity was significantly reduced in spring (*p* = 0.005) and summer (*p* = 0.050) compared to autumn. There was no statistically significant effect of β-GLU activity between the fertilization groups in each season (Figure 5c). In autumn, reduced activity was observed in all groups compared to the C group, but none of the differences were significant. As with NAG, seasonal fluctuations only had a significant effect on β-GLU activity in the C group. In autumn, activity was significantly increased compared to summer (*p* = 0.004), spring (*p* < 0.001) and winter (*p* = 0.002). A statistically significant difference in ARS activity between groups was only observed in summer (Figure 5d). The addition of SF significantly increased the activity of ARS compared to the C group (*p* = 0.005), SMS (*p* = 0.006) and SF.SMS (*p* = 0.007) group. In addition, although not significant, an increase in activity was found in the C group in the fall compared to the other groups. In winter and spring, no significant differences in ARS activity were found between the groups. Here, seasonal changes could only be observed in the SF group. A statistically significant difference was found between summer and winter (*p* = 0.009) and summer and autumn (*p* = 0.031). All types of fertilization had no significant influence on the activity of URE between the groups in all seasons (Figure 5e). However, compared to the C group, some increase in activity was observed in winter and spring and some decrease in summer and autumn. URE activity in all groups also did not appear to be affected by seasonal fluctuations. Compared to the C group, some effect of fertilization on ARN activity was observed in autumn (Figure 5f), although the reduction in activity was not significant. No statistically significant differences were found between any group in the other seasons either. Interestingly, winter had the highest ARN activity in all groups (37.25–40.68 µU/g dry soil). However, as with PHOS (Figure 5a) and URE (Figure 5e), none of the seasons had a significant effect on the activity of this enzyme in all fertilization groups. PAK activity is shown in Figure 5g. Autumn again appeared to be the season with the most enzyme activity in all groups (111.43–126.68 µU/g dry soil) compared to the other seasons. Here, the activity of PAK was reduced after the addition of fertilizers. However, the effect of all fertilizers was not statistically significant. As with ARS activity (Figure 5d), only the SF group was affected by the seasons. A statistically significant difference was found between winter and autumn (*p* = 0.046). As with PAK, the activity of the enzyme PAC (Figure 5g) was also highest in the autumn (43.89–59.66 µU/g dry soil). In addition, all fertilizers reduced the activity in this season compared to the C group. However, none of the differences in PAC activity between the groups were significant in all seasons. As with the first two enzymes, seasonal changes in PAC activity were only observed in the C group. In fact, there was a significant increase in PAC activity in autumn compared to winter (*p* = 0.038) and summer (*p* = 0.014).

- Farm S

**Figure 6:**
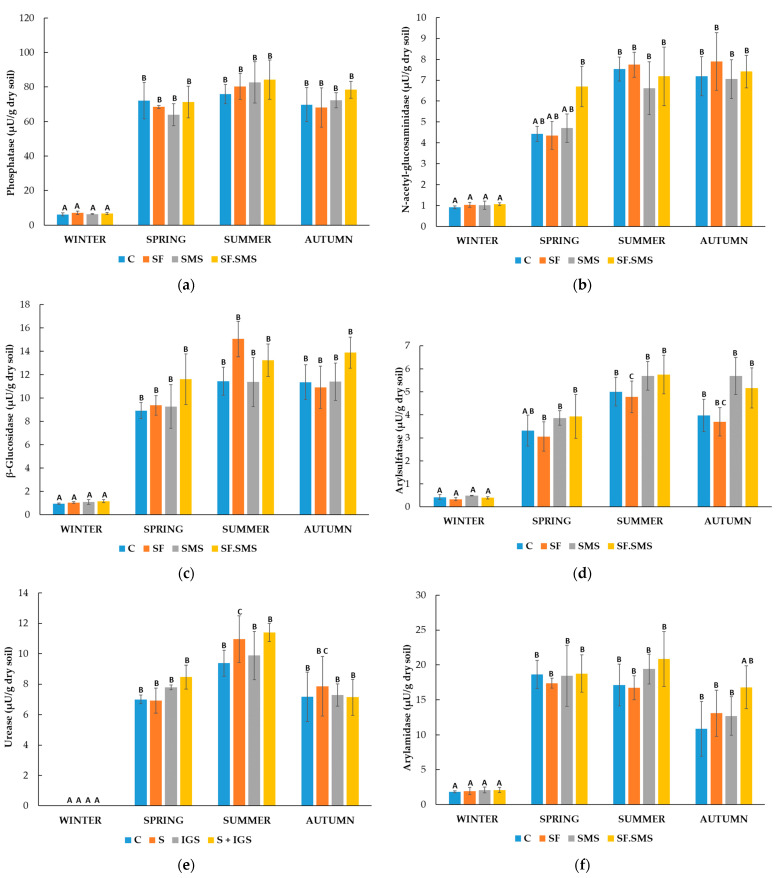

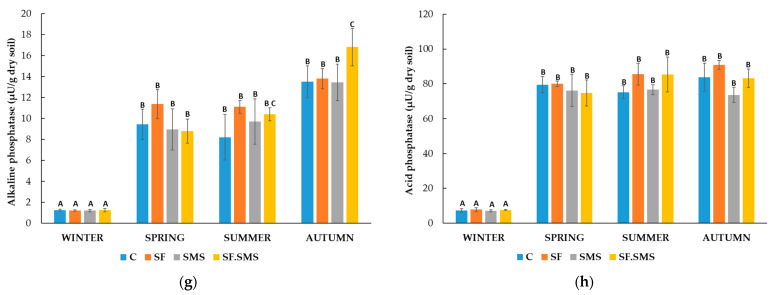
Effect of different fertilization types and seasonal changes in enzymatic activity in farm S. Enzymatic activity of phosphatase (**a**); enzymatic activity of N-acetyl-glucosaminidase (**b**); enzymatic activity of β-glucosidase (**c**); enzymatic activity of arylsulfatase (**d**); enzymatic activity of urease (**e**); enzymatic activity of arylamidase (**f**); enzymatic activity of alkaline phosphatase (**g**); enzymatic activity of acid phosphatase (**h**). Different uppercase letters indicate significant differences between the seasons in the same fertilizer treatment (*p* < 0.05). Data from three independent experiments are presented as medians (geometrical shapes) with ranges. Soil without fertilizer (C: C group); soil with single dose of composted spent mushroom substrate (SMS); soil with single dose of standard fertilization (SF); soil with half dose of SF and half dose of SMS (SF.SMS).

The lowest effect of different fertilizations on PHOS activity was observed in winter (Figure 6a). In addition, a different effect was observed in spring and autumn after different fertilizations. In summer, all types of fertilization increased PHOS activity. However, none of the fertilization effects mentioned were statistically significant. Seasonal changes were observed in the warmer seasons (spring, summer and autumn) for all fertilization groups compared to the C group. In fact, a significant increase in PHOS activity was observed in spring, summer and autumn in the C group (*p* = 0.012, *p* = 0.009 and *p* = 0.010. respectively), SF group (*p* = 0.010, *p* < 0.001 and *p* = 0.033, respectively), SMS group (*p* = 0.012, *p* < 0.001 and *p* = 0.004, respectively) and SF.SMS group (all *p* < 0.001). The activity of the NAG enzyme is shown in Figure 6b. Like in PHOS, the lowest NAG activity was determined in winter (0.91–1.06 µU/g dry soil). In other seasons, only non-significant differences between the fertilization groups were observed. However, seasonal variations showed more effects on NAG activity. In all fertilization groups, a significant increase in the activity of the enzyme was observed in the warmest seasons (summer and autumn) compared to the winter: C group (*p* = 0.019 and *p* = 0.029, respectively), SF group (both *p* = 0.001), SMS group (*p* = 0.049 and *p* = 0.039, respectively) and SMS.SF group (*p* = 0.014 and *p* = 0.017, respectively). In the latest group, a statistically significant difference between the NAG activity in spring and winter (*p* = 0.033) was found as well. A similar trend in the effects of fertilization as in Figure 6a,b was observed for β-GLU activity (Figure 6c). Spring, summer and autumn were again the most active seasons in which β-GLU activity increased the most for all groups compared to the C group. Again, the effects of fertilization were not statistically significant. Nevertheless, statistically significant differences were found between the warmest seasons and winter for all fertilization groups. In fact, β-GLU activity was again significantly reduced in winter compared to spring, summer and autumn for the C group (*p* = 0.013, *p* < 0.001 and *p* = 0.007, respectively), the SF group (*p* = 0.021, *p* < 0.001 and *p* = 0.005, respectively), the SMS group (*p* = 0.001, *p* < 0.001 and *p* < 0.001, respectively) and the combined SF.SMS group (*p* = 0.001, *p* < 0.001 and *p* < 0.001, respectively). Figure 6d shows the activity of the ARS enzyme in each season. The highest activity of ARS appeared to be in summer (4.78–5.75 µU/g dry soil) and the lowest in winter (0.33–0.48 µU/g dry soil), regardless of the fertilization. However, the effect of different fertilization on ARS activity was not significant for all the seasons. Statistically significant increases in ARS activity in spring, summer and autumn, compared to winter were observed in the SF (*p* = 0.001, *p* < 0.001 and *p* < 0.001, respectively), the SMS (*p* = 0.003, *p* < 0.001 and *p* < 0.001, respectively) and the SF.SMS (*p* = 0.002, *p* < 0.001 and *p* < 0.001, respectively) groups. In the SF group, a significant difference in enzyme activity was also found between spring and summer (*p* = 0.035). In the C group, a significant increase in ARS activity was restricted to summer (*p* = 0.018) and autumn (*p* = 0.050) when comparing activity in winter. URE activity is shown in Figure 6e. Again, the highest enzyme activity was detected in the warmer seasons (summer, SF.SMS: 11.38 µU/g dry soil), while no activity of URE was detected in winter. It appeared that all fertilization types affected the activity of URE compared to the C group; however, none of the effects were significant. Due to the inactive winter season, the change to warmer seasons significantly increased the activity of URE in all groups. Statistically significant differences between winter and spring/summer/autumn in the C (*p* = 0.011/*p* = 0.003/*p* = 0.012), SF (*p* = 0.006/*p* < 0.001/*p* = 0.002), SMS (*p* = 0.007/*p* < 0.001/*p* = 0.011) and SF.SMS groups (*p* < 0.001/*p* < 0.001/*p* = 0.001) were found. In addition, URE activity was significantly higher in the SF group in summer than in spring (*p* = 0.030). The highest activity in ARN was observed in all groups in spring and summer, followed by autumn and winter (Figure 6f). Fertilizers slightly increased the enzyme activity compared to that of the C group in spring, summer and autumn. However, this was not the case with SF in spring and summer. Nevertheless, the differences between the fertilization groups were not significant. As expected, the reduction in ARN activity in winter compared to the other seasons (spring, summer and autumn) was significant in the C group (*p* < 0.001, *p* < 0.001 and *p* = 0.014, respectively), the SF group (*p* < 0.001, *p* = 0.008 and *p* = 0.016, respectively) and the SMS group (*p* = 0.001, *p* = 0.001 and *p* = 0.020, respectively). In the SF.SMS group, a decrease in ARN activity in winter was only observed when comparing activity in spring and summer (*p* = 0.040 and *p* = 0.023, respectively). Spring, summer and autumn were again the most active seasons in terms of PAK activity (Figure 6g). In contrast to other enzymes, the highest PAK activity was observed for all groups in autumn. Except in spring for the SMS and SF.SMS, fertilization increased PAK activity compared to the C group. In all seasons, however, the differences between the fertilization groups were not significant. The effect of seasonal variation could be detected in all groups as PAK activity was low in winter (1.20–1.24 µU/g dry soil). Indeed, PAK activity increased significantly in all groups in spring, summer and autumn compared to the activity in winter (group C: *p* = 0.007, *p* = 0.016 and *p* < 0.001, respectively; SF: *p* = 0.018, *p* = 0.003 and *p* = 0.006, respectively; SMS: *p* = 0.003, *p* = 0.002 and *p* < 0.001, respectively; SF.SMS: *p* = 0.015, *p* = 0.010 and *p* < 0.001, respectively). A statistically significant difference was also found between the activity in spring and autumn in the SF.SMS group (*p* = 0.030). Figure 6h shows PAC activity. Only small non-significant differences in PAC activity were seen between all fertilization groups in each season. In fact, the activity of this enzyme was quite stable when comparing the warm seasons (spring, summer and autumn). As for all previously mentioned enzymes, PAC was the least active in winter in all groups (7.08–7.84 µU/g dry soil). Not surprisingly, the activity was decreased significantly compared to the activity in spring, summer and autumn (C group: *p* < 0.001, *p* = 0.001 and *p* < 0.001, respectively; SF: all *p* < 0.001; SMS: *p* = 0.018, *p* = 0.001 and *p* < 0.044, respectively; SF.SMS: all *p* < 0.001).

## 4. Discussion

### 4.1. Effect of Different Fertilization Types and Seasonal Changes on the Microorganism Quantities

A special feature of actinomycetes is that they produce several enzymes and they can use them to degrade complex organic materials in soil and sediment, especially some poorly degradable insect and plant polymers such as chitin, cellulose, hemicellulose and other organic materials [22]. Therefore, the higher the value of these microorganisms in the soil, the better, as they have a positive effect on the soil structure. Actinomycetes can also produce organic acid and play an important role in nitrogen fixation and the production of sideophores [34]. As part of the phylum Actinomycetota, they have aroused the interest of the agricultural industry as a source of biologically active compounds, biocontrol agents, plant growth-promoting bacteria and plant growth regulators [35,36]. The secondary metabolites produced by these microorganisms are also used for pharmaceutical and food purposes [21,37]. In general, a 10-fold higher number of actinomycetes (1–3 × 10^7^ CFU/g dry soil) was found in the soil in farm T (Figure 2a) compared to the lower number (2–8 × 10^6^ CFU/g dry soil) in farms M (Figure 1a) and S (Figure 3a). These results were to be expected as farm T is a biodynamic farm where management approaches are geared towards greater microbial diversity and higher abundance, due in part to a rich soil structure (organic matter and carbon content—Table 2) [38,39,40]. Moreover, actinomycetes represent a large group of soil microorganisms and are known as important decomposers of poorly degradable organic matter [22]. Furthermore, although organic farming leads to a considerable amount of organic matter and carbon in soil, the number of actinomycetes in farm S (Figure 3a) was generally lower, most likely due to the rather low soil pH (4.2) and the fact that this group of microorganisms is relatively sensitive to acidity. In fact, their optimal pH range is between 6.5 and 8.0 [34]. Irrespective of fertilization, seasonal changes had a relatively mild influence on the number of actinomycetes in three farms. Statistically significant differences were only observed between winter and the warmer seasons in farm M (Figure 3a) and farm S (Figure 5a). In farm T (Figure 4a), on the other hand, seasonal fluctuations had no influence on the number of actinomycetes. Our results are not surprising, as there are known reports of actinomycetes (psychrophilic to thermophilic strains) being able to grow in a wide temperature range (4–60 °C), with optimal temperature growth at 28 °C (reviewed in [34,37]). Studies have reported that mineral fertilizers increase the number of actinomycetes [41,42,43,44] and Actinomycetota [45,46] due to the increased availability of nitrogen and the fact that a lot of actinomycetes are free-living diazotrophs and therefore play an important role in nitrogen fixation and its supply to ecosystems [34,35]. In our study, SF in spring in farm M (Figure 3a) and in autumn in farm S (Figure 5a) significantly increased the number of actinomycetes compared to C and some other fertilizer types. In the other seasons, however, the effect of SF was not significantly different compared to other fertilization groups. Moreover, SF in farm T (Figure 4a) decreased rather than increased the number of actinomycetes (except in spring). This is probably due to the greater diversity and quantity of microorganisms in biodynamic soils [38], which leads to competition between microorganisms as organic decomposers and suppression of actinomycete growth. Namely, actinomycetes have a lower activity and growth capacity than other bacteria and fungi [34]. Supplementation with SMS significantly increased the number of actinomycetes in farm M (autumn), farm T (spring and summer) and farm S (summer and autumn). Since SMS is an organic and, therefore, a slow-release compound fertilizer due to its structure (calcium carbonate) [9,47], it is not surprising that it has a positive effect on actinomycetes. In addition, the structure of composted SMS consists, among other additional nutrients, of less compostable organic matter, a substance that is favorable for the activity and growth of actinomycetes [9,34,47], as well as fungi [48,49,50,51,52]. In fact, SMS has already been shown to positively influence the growth of certain bacteria (including strains belonging to the phylum Actinomycetota) and fungi [48,53,54,55]. Even in forest soils, *Pleurotus* and strains of fungi such as *Stropharia rugosoannulata*, have been shown to positively influence both numbers and microbial richness, leading to greater active functioning [56]. An interesting observation was noticed at the combined fertilization (SF.SMS) in all three farms, especially in the seasons when SMS and/or SF alone had a poor or negative effect on the number of actinomycetes (Figure 3a spring, Figure 2a autumn, Figure 1a summer). The addition of a combined fertilizer seemed to enhance the effect of both fertilizers, resulting in a better (Figure 1a summer and Figure 2a autumn) or even significantly greater (Figure 3a spring) effect on the number of actinomycetes. There are reports in the literature stating that a low dose of mineral fertilization helps to stimulate the decomposition of organic matter, which leads to an increase in the amount of organic matter in the soil [57,58,59,60], and that the combination of organic and mineral fertilizer is a good substitute for nitrogen fertilization in this respect [61]. Moreover, it could be suggested that the higher counts of actinomycetes in enriched SF with organic SMS are due to sensitivity for organic compounds in combined chemical/organic fertilizer [62,63,64]. 

Besides bacteria, soil fungi play an important role as biological controllers [65,66,67], ecosystem regulators [23,68], and decomposers of organic material or transformers of compounds [69]. They produce different types of extracellular enzymes that convert organic matter into biomass, organic acid and CO_2_, and simultaneously decompose soil components to maintain nutrient equilibrium [13,70]. When comparing the number of soil fungi in all three farms (Figure 1c, Figure 2c and Figure 3c), it is not surprising to find that farm T, followed by farms S and M, is the most densely populated with fungi. We found approximately 10^6^–10^7^ CFU of fungi/g dry soil in farms T and S, which was at least 10 × higher than in farm M. It is known that both biodynamic and organic farming promote soil microbial diversity and abundance [39,40,71,72,73] and are positively correlated with organic matter and total carbon [38,40,71,74,75]. In fact, both organic matter and carbon content were higher in farms T and S than in farm M (Table 2). Seasonal changes could also be observed in the number of fungi in all three farms. It is already known that soil microorganisms, including fungi, can directly depend on soil moisture and temperature [2,23,51,76]. This was also evident in our study, in which autumn and summer were the seasons with a significantly higher number of fungi compared to winter and spring in all three farms. In addition, autumn and summer also showed significant effects on fungi between fertilizer groups in all three farms. In farm M (Figure 1c), the largest and most significant effect of SF on fungi count was observed in summer compared to the other two fertilizations, but not to the C. It is known that mineral fertilizers lower soil pH [51,60,71,74,77,78] and that fungi can adapt more easily to acidity [2,23,24,51,74]. However, this could also partly explain the highest but non-significant effect of combined fertilization on fungi number in autumn, which has also been confirmed by other studies [51,79,80]. A similar, significant effect of SF and SF.SMS on fungi was observed in farm S, in both seasons (Figure 3c). SMS fertilization also had a significant effect on the number of fungi. In fact, the number of fungi in farm S (Figure 3c) was the highest in summer and winter due to SMS fertilization. A significant increase in the number of fungi upon SMS fertilization compared to the other groups was also observed in farm T (Figure 2c) in summer. A positive effect of composted SMS due to its enriched organic composition (Table 2) on the physical, chemical and microbiological properties of soil [51,56,60,81,82], as well as on fungal development and structure [48,49,50,51,56,83], has already been reported. In addition, our results could also confirm a reported fact that the SMS has a better ability to retain water in the soil, the amount of which is known to be lower in winter and summer, resulting in a better response of microorganisms and plants to drought [60]. Although the number of fungi in autumn in farm T was still very high compared to the number in other seasons, a significant decrease after all fertilization types compared to the C group was observed. We have no explanation for this phenomenon, as for the biologically active structure typical of biodynamic soils and the suitable climatic conditions (autumn) for the optimal growth of fungi in this soil type [38,84].

As expected, farm T (Figure 2b) had a 10-fold higher total number of microorganisms (1–2.5 × 10^7^ CFU/g dry soil) compared to farms M (Figure 1b) and S (Figure 3b), regardless of the season, which is, in turn, related to the high microbial diversity and abundance in soil typical of biodynamic farming [39,40,71,72,73]. The highest number of total microorganisms was observed in autumn, followed by summer, which was statistically significant for almost all the groups, compared to the number in cooler seasons (winter, spring). It is already known that the activity and growth of microorganisms are directly dependent on soil and ambient temperature and moisture [2,51,85]. Interestingly, the highest number of microorganisms was detected in the fall in group C, which was also statistically significant and could be directly correlated with the highest number of fungi detected in the same season (Figure 2c). The seasonal trend was also observed in farm S (Figure 3b), but not completely in farm M (Figure 1b). Compared to the other two farms, the summer number of total microorganisms was very low, which could be partly related to the low moisture content in the soil (Table 2) and the fact that most microorganisms are very sensitive to the availability of free water that binds the soil particles [71]. In addition, the only statistically significant effect was observed by fertilization in spring (Figure 1b). Supplementation with SMS led to a statistically significant increase in the total number of microorganisms. Apparently, the organic-rich structure of SMS and its ability to retain more water in the soil [60] favored the growth of microorganisms in spring. In farm T (Figure 2b), a statistically significant effect of SMS on the amounts of actinomycetes and fungi in summer apparently led to a statistically significant increase in the number of total microorganisms. Moreover, the addition of SMS also led to a significant increase in the number of total microorganisms in farm S in summer (Figure 3b). The addition of SMS possibly stimulated the soil core microflora [81], or the increased total number was due to an input of waste microflora [86,87], or even both, which could also be observed in other soil types and positively influenced the structure of the bacterial and fungal community [56]. In farm S, a significant increase in the total number of microorganisms (Figure 3b) was also observed in summer after the addition of SF, probably due to a significant increase in the number of fungi (Figure 3c). Few studies reported a significant increase in the total number of bacteria and fungi after chemical fertilization, emphasizing the importance of choosing the right fertilizer dose [42,43,44,73,88]. 

### 4.2. Effect of Different Fertilization Types and Seasonal Changes on the Enzyme Activity in Three Farms

In general, a comparison of the enzyme activity in all three farms (Figure 4, Figure 5 and Figure 6) showed that farm M (Figure 4) had the least enzymatically active soil. These results are probably due to conventional–integrated farming, where, besides basically used organic fertilizers, constant use of mineral fertilizers affects soil structure and the quantity, diversity and enzymatic activity of soil microorganisms [39,71,89]. In addition, soil moisture content has been shown to be one of the most important factors affecting microorganisms, their diversity and activity [71]. In fact, the soil of farm M had the lowest overall moisture content (Table 2), which could also explain the lowest number of microorganisms, already previously observed, and total enzyme activity. 

The amount of soil phosphorus available to plants influences the early growth of plants and is essential for all living organisms [90]. In soil, phosphorus occurs in organic, inorganic and soluble forms, with organic phosphorus trapped in organic matter and organic residues being immutable and structurally inaccessible to plants. The mineralization of phosphorus is carried out by microorganisms that produce phosphatases. With the help of these enzymes, organic phosphorus is converted into phosphate, a form that can be taken up by plants [91]. PHOS activity is used as an indicator of the availability of inorganic phosphorus in the soil for plants and microorganisms and is an important determinant of soil quality [27]. The main sources of PHOS in soil are microorganisms and plant roots, as the amount of phosphatases in soil varies depending on the amount and diversity of microbial composition and organic matter [92]. The activity of PHOS in farm T and farm S (Figure 5a and Figure 6a, respectively) was approximately 2 times higher than the one in farm M (Figure 4a). Farm T acts according to the guidelines of biodynamic agriculture, which rejects the use of synthetic pesticides and mineral fertilizers and promotes the use of natural substances such as food waste or compost, resulting in soil that is consequently richer in nutrients and organic matter [93,94]. Therefore, the increased enzyme activity could be due to the fact that the sampled soil was already fundamentally richer in enzymes. Organic farming in farm S revealed a higher amount of organic matter as well (Table 2), compared to farm M. Furthermore, the amount of PHOS in the soil is also influenced by the use of mineral and organic fertilizers and tillage techniques [92], which could explain the lowest PHOS activity in farm M (Table 1) and the different effects of tested fertilizers observed in all seasons and in all farms, none of which were statistically significant compared to the C group. According to our results, seasonal changes did have a statistically significant impact on the activity of PHOS in farms M (Figure 4a) and S (Figure 6a), but not in farm T (Figure 5a). The activity of PHOS in farm T was constant during all seasons in all fertilizer groups, with a little reduction in winter. This fact could be due to the cultivation and treatment techniques of the soil [95], the high proportion of organic matter and the fact that the enzymatic activity of the soil is closely related to the soil microbiota, which is typical of the biodynamic method of farming [93]. Cao et al., 2021 [96] explained that seasonal variations in phosphatase activity are due to changes in ambient temperatures, rainfall and soil conditions. Furthermore, PHOS activity is affected by the weather and thermal factors [97], which could explain the reduced activity in winter and higher activity in warmer seasons in farms M and S (Figure 4a and Figure 6a, respectively).

Phosphomonoesterase, including acid and alkaline phosphatase (PAC and PAK, respectively), is the most active and best-studied phosphatase in soil. The availability of phosphatases depends strongly on the pH value of the soil. PAC activity is mainly present in soils with pH values between 4 and 6, while PAK dominates in basic soils where the pH is between 9 and 11 [98]. Our results could confirm the effect of pH on the activity of PAK and PAC, especially in farm S (Figure 6g,h), where the pH of soil was 4.2 (Table 2), resulting in the lowest activity of PAK and the highest activity of PAC, compared to the other two farms (Figure 4g,h and Figure 5g,h). The soil in farms M and T had a pH of approximately 7 (Table 2), resulting in higher activity of PAK than PAC. The highest PAK activity in farm S (Figure 6g) was determined in autumn, in group SMS.SF, which could be due to the combination of mineral fertilization [92,99] and the late release of organic matter from the spent mushroom substrate [84]. However, the addition of fertilizers did affect PAK by increasing its activity in all warmer seasons, yet none of the effects were significant. Furthermore, autumn was the season with the highest PAK activity in farms M and T (Figure 4g and Figure 5g, respectively) as well. In fact, in farm M (Figure 4g), the biggest effect on PAK activity was due to SMS fertilization. Cao et al., 2023 [96] stated the amount of organic matter in the soil, the composition of the microbial communities, the depth of soil sampling and the ecological succession of the soil as the most important factors influencing the activity of phosphatases. Since SMS is rich in organic matter and nutrients (Table 2), its decomposition by microorganisms could cause the values of available phosphorus and phosphatase activity in the soil to be higher in the fall months than in the spring and summer, when the decomposition rate is lower [9,27,91]. PAK activity also depends on the pH of the environment in which it is located [98], so the addition of SMS could help to create optimal, slightly alkaline conditions (Table 2) for its activity [51,98]. Among all the farms, farm T had the highest PAK activity in all seasons (Figure 5g). Interestingly, the highest activity was determined in the C group (autumn). Adetunji et al., 2017 [27] stated that the activity of enzymes from the group of phosphatases, including alkaline phosphatase (PAK), is an indicator of soil quality, indicating a general diversity in the composition and structure of biodynamic soils [38]. The latter could dominate the effects of the added SMS in the form of fertilizer, as PAK enzyme activity in SMS and SF.SMS was comparable throughout the year. The lowest enzyme activity was found in winter in all three farms, which is consistent with the results of a two-year study by Touhami et al., 2022 [100], who reported the effects of low temperatures on the reduction in phosphatase activity in soil. Unlike in other two farms, farm T had a non-significant difference between the activity of PAK in winter and other seasons, most likely due to the variety in the biodynamic soil structure [38]. The activity of PAC enzyme in farm M (Figure 4h) was significantly increased in the SMS group compared to the C group only in spring. Since PAC hydrolyzes organic phosphorus compounds and subsequently converts them into various forms (inorganic phosphorus) that can be directly utilized by plants [101], its highest activity in spring could be due to the richest composition of organic matter in the SMS [102], including organic phosphorus. However, the activity was not significantly decreased in this group in the following seasons, which could be due to the relatively stable composition of SMS, resulting in the slow mineralization of the organic matter as a consequence of composting or fermentation processes of the SMS preparation [81]. In addition, the influence of the pH of SMS over time could also have an inhibitory effect on the activity of PAC, as the average measured pH of the mushroom substrate is 7.3 [103] (see Table 2) and it is typical for PAC to be optimally active in an environment with a pH between 4 and 6 [98]. Furthermore, autumn was the most active season for PAC in all three farms as well. In this season, however, all fertilizations reduced the activity of PAC in farm T (Figure 5h), compared to the C group. The results could point to a high level of phosphorus mineralization due to high enzyme activity in the autumn months [91], but the reduction in activity after fertilization could again be due to the higher pH [103]. The pH of soil in farm S was 4.2, resulting in higher activity of the PAC enzyme in all warmer seasons (Figure 6h), which is in concordance with Banerjee et al., 2012 [92]. The results also showed a tiny effect of different fertilizations with SF increasing the activity of PAC at most, which can confirm also the study of Sawicka et al. [104], reporting a significant effect of mineral fertilization on the PAC activity. Symanowicz et al., 2022 [99] reported on the negative impact of the use of mineral fertilizers on acid phosphatase activity in the soil, which is the opposite. Such a result suggests the possibility that there were other factors that influenced the activity of PHOS in this case, so it would be necessary to carry out further research for a clearer interpretation of these results. A significant reduction in PAC activity was observed in winter, compared to the other three seasons in farms M and S (Figure 4h and Figure 6h, respectively). Touhami et al., 2023 [100] investigated the influence of temperature on the activity of various phosphatases in soil and reported similar results. Interestingly, the PAC activity in farm T (Figure 5h) was fairly constant during most of the year, regardless of the fertilizations, which in turn could indicate that a high concentration of PAC is already present in biodynamic soil [38].

Between 5 and 25% of carbohydrates are found in soil organic matter, mainly in the form of simple sugars, hemicellulose, cellulose and other remnants of plant matter, which are then decomposed by microorganisms and used for the synthesis of more complex molecules [105]. Glycosidases are a group of enzymes that catalyze the hydrolysis of glycosides, which are the main energy source for soil microorganisms. Among the glycosidases, α-glucosidase and β-glucosidase (β-GLU) as well as α-galactosidase and β-galactosidase are most abundant in soil [27,52]. The activity of glycosidases has been positively correlated with soil pH in the past and their activity has been found to increase with increasing soil organic carbon [98]. NAG is an important glycosidase enzyme involved in the degradation of chitin and other β-1,4-linked glucosamine polymers to simple N-acetylglucosamine units. NAGs are mainly produced by fungi that contain chitin in their cell walls. Hydrolysis processes that occur under the influence of NAG enable the cycling of carbon and nitrogen [106]. By comparing the three farms (Figure 4b, Figure 5b and Figure 6b), the NAG activity in farm M (Figure 4b) was found to be the lowest, which could be explained by the use of mineral fertilizer as additional fertilization. In fact, the literature shows that high N and P fertilizer applications can suppress NAG activities, leading to a decrease in enzyme production when inorganic nutrients are readily available [107,108]. Moreover, NAG activity in farm M was detected only in winter, where the addition of SMS significantly increased the activity of NAG compared to the SF, but not compared to the C group and SF.SMS. In fact, higher NAG activity has been positively associated with soil pH and the amount of organic carbon and nitrogen in the soil [98,102], both present in high amounts in SMS and higher-plant-derived substrates as well [106]. Moreover, NAGs are mainly produced by fungi, and higher NAG activity may also indicate a higher microbial turnover in their favor [109]. A similar trend after the addition of different fertilizers and therefore a similar explanation could be observed in farm T in autumn (Figure 5b). However, compared to the very high NAG activity in the C group in this farm, the addition of fertilizers reduced its activity significantly. Compared to other groups, a relatively high NAG activity in the C group could reflect a high amount of fungi (Figure 2c). It is not surprising that the highest (except in winter), mostly consistent activity of NAG between the groups was found in farm S (Figure 6b). A relatively low pH accelerates the leaching of nutrients from the soil, which exacerbates nutrient limitation for soil microbes, hence causing a potential increase in NAG activity, whose hydrolysis processes enable the cycling of carbon and nitrogen [106]. 

β-GLU activity is the most commonly used indicator of soil quality, which is closely linked to soil organic matter, biological activity and carbon cycling [98]. The activity of β-GLU was relatively high throughout all seasons in farm T (Figure 5c), with the highest value measured in autumn in the C group and the lowest in the spring, also in the C group. This result was to be expected, as β-GLU is one of the most abundant enzymes in nature, enabling carbon cycling, among other things. High β-GLU activity in soil is generally associated with increased soil organic matter content [51,110], a characteristic of biodynamic soils, which are often more biologically rich compared to conventional farming methods [38]. However, in this case as well, high activity in the C group in autumn may be linked to the high amount of fungi (Figure 2c), as β-GLUs are widely produced by different genera and species of the fungal kingdom including Ascomycetes and Basidiomycetes [111]. Moreover, in the SMS and SF.SMS groups, the relatively constant activity of β-GLU in spring and summer followed by the high activity in autumn could indicate a slower release of organic matter from the SMS. Sun et al., 2021 [84] found that, besides high temperatures, the addition of exogenous enzymes for the degradation of the biological mass could lead to faster degradation of the spent mushroom substrate, which was not the case in the biodynamic soil. Farm M (Figure 4c) had the lowest activity of β-GLU (4–10 times), compared to farms T (Figure 5c) and S (Figure 6c), which reflects the higher content of organic carbon in ecological and biodynamic farming (Table 2). Nevertheless, the enzyme β-GLU was most active in the summer months, where the highest level was measured in the SMS group. Uwituze et al., 2022 [98] reported that β-GLU activity is among the most commonly used indicators of soil quality, reflecting soil organic matter content, soil biological activity and carbon cycling. The high activity of β-GLU in the groups SMS and SF.SMS could also be attributed to the rich composition of SMS, which contains high amounts of biologically active compounds, lignocellulose and enzymes, among others [112]. The β-GLU activity decreased in the C group and in the SMS during autumn and winter months, while its concentrations remained slightly higher in treatments with the SF and the SF.SMS. Further studies would be necessary to determine whether the increased activities were caused by the addition of mineral fertilizers, since data from the literature suggest a link between increased activity of glucosidases and the use of nitrogen mineral fertilizers [113]. The activity of β-GLU in farm S (Figure 6c) was similar to that in farm T (Figure 5c), except for winter, where the activity was significantly reduced, compared to other seasons. This indicates that enzymatic and microbiological activity, along with the amount of organic matter in soil, is reduced but still present in the colder months [114]. Soil in farm S was relatively acidic. However, our results show that β-GLU activity is not sensitive to an acidic environment. The optimal pH for most β-GLUs is between 4.0 and 7.5, and they tend to be stable in a pH range of 4.0 to 9.0 [52]. The activity of β-GLU significantly increased in spring and reached its maximum activity in summer in the SF and SMS.SF groups. The increase in enzyme activity in the group where standard fertilization was applied could indicate a link between using mineral fertilizers and increased activity of glucosidases in soil [113]. In the fall months, β-GLU activity decreased in all groups, with the exception of the SF.SMS group. The reason could be the slower degradation of organic matter from the SMS [81,84], which could cause the value of β-GLU to remain elevated, rather than the addition of standard fertilizer, as a decrease in enzyme activity is also observed in the group where only standard fertilization was applied (SF).

Most soil sulfur is in organic form and accounts for 90–98% of total sulfur. The predominant organic form of soil sulfur is sulfate ester, which accounts for 30–75%. Mineralization of organic sulfates is necessary for sulfur availability in soil [115]. ARS belongs to a subgroup of sulfatases and catalyzes the hydrolysis of organic sulfate esters to free sulfonate groups [116,117]. These enzymes are mainly produced by fungi and bacteria, but to a lesser extent by lower plants and animals [118]. ARSs synthesized by natural microorganisms work optimally in the temperature range between 20 and 57 °C [118], which was observed in farms M and S (Figure 4d and Figure 6d, respectively). ARS activity was highest in the summer and autumn months, regardless of the fertilization type, and reached its lowest values in winter. Nevertheless, the latter was not the case in farm T (Figure 5d), where ARS activity was relatively stable throughout all seasons, except for the significant increase after SF addition in summer, compared to the other three groups. The reason for this phenomenon is unknown, as ARS activity in other seasons was the lowest after SF in comparison to other fertilizer groups. SF also decreased the activity of ARS in the other two farms, compared to the C and other fertilization groups (Figure 4d and Figure 6d). The use of mineral fertilizers has an inhibitory effect on ARS activity [60,105]. The lowest activity of ARS was again detected in farm M (Figure 4d), when compared to that in farms T and S (Figure 5d and Figure 6d, respectively), which could be attributed to the sensitivity of this enzyme to the type of farming, crops grown, organic matter content and soil fertilization [105]. However, in farm M (Figure 4d), significant differences in ARS activity between the groups were determined in spring. A significant activity decrease in SF and SF.SMS could be attributed to the inhibitory effect of the mineral fertilizer [105]. The optimal pH range for the activity of acidic and basic arylsulfatases is between 6.5 and 7.1 and between 8.3 and 9.0, respectively [118]. Nevertheless, ARS was apparently also relatively active in lower pH ranges (Figure 6d), most likely due to the organic fertilization (Table 2). Moreover, a higher enzyme activity of ARS in the SMS and SF groups in warmer seasons could be observed, compared to the SF and C groups, which could be attributed to the partial decomposition of organic matter from the spent mushroom substrate [84].

One of the most important components for plant growth, which is also an indicator of soil health, is nitrogen. Nitrogen is present in soil in four main forms: (a) as ammonium ions (NH_4_^+^), retained by organic matter and clay minerals, (b) as mineral nitrogen (NO_2_^−^, NO_3_^−^, NH_4_^+^), (c) in microbial and clay biomass, and (d) in organic matter, fungi, plant material and humus [119]. URE in soil is mainly of microbial and plant origin and acts as an intracellular and extracellular enzyme. It acts by hydrolyzing urea into ammonium ions (NH_4_^+^) and carbon dioxide (CO_2_), which leads to an increase in soil pH [27]. Over the years, research on URE activity in soil has shown that URE is a good indicator of soil quality due to its role in regulating nitrogen supply to plants after urea fertilization [120,121]. Statistically significant seasonal variations affecting URE activity could be observed in farms M and S (Figure 4e and Figure 6e, respectively), regardless of the fertilization type. The activity of URE started to increase in the spring months and reached the significantly highest values in summer, followed by autumn. The high activity of URE in the warm season and low activity in winter are consistent with the results of some studies [27,120], which state that the stability of urease depends on many factors, including soil moisture and temperature, and that the urease activity in soil increases with increasing temperature in the environment and the soil and vice versa. The relatively constant activity of URE in farm T (Figure 5e) throughout all the seasons could be attributed to the stability of the soil structure due to the positive influence of biodynamic farming on the quality and diversity of soil, compared to the conventional farming methods [38]. Interestingly, the highest activity of URE in farm T, although not significant, was detected in winter after SF treatment. The relationship between URE activity in soil and the use of mineral fertilizers was also reported by Jabborova et al., 2021 [122], who found an 87% increase in URE activity when the soil was treated with mineral fertilizer compared to the C group without fertilizer. This effect was also observed in farm S (Figure 6e), but not significantly different from the other fertilizer groups. In the warmer seasons (summer, autumn), a relatively comparable URE activity was observed in all farms between the SF group and the groups with added SMS. The addition of organically enriched SMS could partially support the URE activity, especially in the fall months, as the decomposition of organic matter in the soil is highest at this time [123] and a large proportion of the total nitrogen in the soil is bound in organic compounds [124].

Besides URE, ARN activity is associated with microbial nitrogen uptake, since the enzyme plays an important role in the initial reaction of N mineralization in soils involved in the release of amino acids from soil organic matter [124,125,126]. In farm T (Figure 5f), ARN activity was present throughout the year, with the highest values measured in winter, and was comparable for all the groups. ARN activity is highly related to nitrogen mineralization in the soil [126], which is a characteristic of biodynamic soils rich in organic matter [38]. The optimal nitrogen mineralization occurs at temperatures above 25 °C [126], which is consistent with observations in the spring and summer months, but does not explain why mineralization activity is highest in the winter months when ambient temperatures are lowest. We see the possibility of such results in the rich microbiological diversity of biodynamic soils [38] and in the slow mineralization of organic matter in SMS [84]. The highest values of ARN activity in farms M and S (Figure 4f and Figure 6f, respectively) were measured in spring and summer and were comparable for all soil treatment methods. The results indicate a high mineralization of nitrogen in soil during these months. In winter, the enzyme activity decreased significantly in all soil treatment methods, which could be attributed to the decrease in ambient temperatures [127]. In farms S (Figure 6f) and M (Figure 4f), ARN activity started to decrease in autumn, where only the SF.SMS group showed a slightly increased activity, indicating the influence of combined mineral and SMS on the prolongation of the mineralization process in soil compared to the other treatments. There is a lack of literature in this area, so further research would be necessary. The results of the experiment could also be influenced by less-known factors, such as the previous use of organic fertilizers or specific needs of tomatoes planted in the area. In fact, Grunert et al., 2019 [128] investigated the influence of the root system of tomatoes on soil microbial communities and concluded that the choice of plants planted has a strong influence on the microbial communities developing in the soil and subsequently soil processes, including the activity of soil enzymes.

## 5. Conclusions

The relationship between soil microorganisms and the soil itself is complex. The diversity of our results shows that initial soil properties have a considerable influence on the number of different microorganisms and their enzymatic activity. Overall, the comparison of all three farms showed that the soil of the biodynamic farm had the highest number of actinomycetes, fungi and total microorganisms in general, regardless of the type of fertilization, followed by the soil of the organic and finally the conventional–integrated farm. In connection with the latter observations and the basic structure of the soil, one might expect a more similar comparison between the biodynamic and the organic farm, but we suspect that discrepancies are mostly due to the low pH of the soil in the organic farm. The use of different fertilizers had some effect on the number of different microorganisms, with the soil in the biodynamic farm appearing to be the least affected. The addition of SF had the greatest effect on the number of fungi, probably due to the acidic effect of the fertilizer. Unlike in farms M and S, the addition of SF reduced the number of actinomycetes in farm T. Nevertheless, the effect of this fertilizer on the number of total microorganisms clearly showed the importance of the soil structure and the dosage of the fertilizer, as the combination of SF and SMS increased the number of soil microorganisms, at least actinomycetes and fungi. The addition of SMS had a relatively positive effect on the number of microorganisms in all farms, which led to higher activity of the microorganisms regardless of the basic structure of the soil. However, it should be kept in mind that a high total number of microorganisms does not always indicate good soil condition, but rather microbial composition, among other complex things, that determines the health of the soil.

The activity of soil enzymes reflects the activity of various organisms responsible for their production, as well as the environmental (abiotic) conditions that directly affect soil, causing the complexity of this relationship. Despite the different involvement in the cycling of elements in the soil, some enzymes (PHOS, PAK, β-GLU, ARS, URE, ARN) clearly showed that their activity was influenced by the structure of the microorganisms and the soil itself, especially its organic matter and C-N-P cycling. Therefore, enzyme activity was obviously higher in biodynamic and organically managed soils. However, the important role of pH on enzyme activity was seen for some enzyme activities, especially PAK and NAG, where the acidic soil of the organic farm reduced the activity of both enzymes. In contrast to PAK and NAG, soil pH does not seem to have a particular effect on the activity of β-GLU, URE and ARS. As expected, supplementation with different fertilizers had contrasting results on different enzymes. PHOS was affected by all fertilizers, but the effects could be more pronounced in the activity of PAK and PAC separately. However, the addition of SF showed some contradictory results, which were reported in other studies as well. Supplementation with SF had the strongest effect on NAG, β-GLU and ARS activity, mainly in farms M and S, but not in T. The stability of soil structure and microorganisms in farm T clearly reflected the stability of enzyme production. Supplementation with SMS and its rich structure had an overall positive effect on increased production of several enzymes (PAK, PAC, NAG, β-GLU, ARS, URE) regardless of the basic soil composition, with the activities of some enzymes (PAK, URE) additionally influenced by the season (autumn). In contrast to actinomycetes and fungi, the combination of SF and SMS seems to increase the overall activity of β-GLU and ARN but has no particular effect on other enzymes.

A seasonal dynamic between warmer and colder seasons was observed in the number of actinomycetes and even more in the number of fungi, showing the important role of environmental factors such as temperature and moisture. Similar to the number of microorganisms, the activity of PAK and ARN decreased in colder seasons in all three farms. However, the activity of PHOS, PAC, β-GLU, ARS and URE decreased only in farms M and S in colder months. This shows that temperature fluctuations seem to have less or no influence on these enzymes in biodynamic farm T, as their activity remained relatively stable throughout the year.

Soil function and health are clearly dependent on its properties, environmental conditions and microbial dynamics. The latter not only change the soil structure but also influence their own activity. Fertilization can influence the number of microbes and their composition, most likely through changes in soil properties, as partially observed in our study. Nevertheless, more comprehensive studies on microbial diversity and their relation to the enzymes after different fertilizer exposures in different agricultural soil types are foreseen.

## Figures and Tables

**Table 1:** Characteristics of the experimental locations.

Characteristic	Farm M	Farm S	Farm T
Location site	46°30′34.7″ N 15°44′08.3″ E	46°24′54.2″ N 15°39′12.1″ E	46°37′52.4″ N 15°41′50.8″ E
Type of farming	Conventional–integrated	Organic	Biodynamic
Standard fertilization	Mineral fertilizer	Commercial fertilizer suitable for organic farming	Own fertilizer (compost)
Basic	Organic fertilizer “Bioorganik”	Pelleted manure	Compost/sunflower seed cake
Additional	Mineral fertilizer “Rosasol K”	None	None

**Table 2:** Properties of the soil in three farms and the SMS.

Parameter	Farm M	Farm S	Farm T	SMS
pH	7	4.2	7.1	7.6
Moisture	17.1	28.1	26.3	84.4
P_2_O_5_ (mg/100 g)	21	19.5	23	345
K_2_O (mg/100 g)	12	17.5	30.5	2015
Mg (mg/100 g)	32	14	37.5	102
Ca (mg/100 g)	446	48	557	987.5
Total N (%)	0.16	0.20	0.31	1.52
N-NH^+^_4_ (mg/kg)	1.2	0.7	0.5	48
N-NO^−^_3_ (mg/kg)	144.6	1.0	60.6	0.5
Total organic C (%)	1.6	2.1	3.4	32.4
Organic matter (%)	2.7	3.5	5.9	58

## Data Availability

The raw data supporting the conclusions of this article will be made available by the authors on request.

## Supplementary Materials

The following supporting information can be downloaded at https://www.mdpi.com/article/10.3390/microorganisms12081521/s1: Table S1: Composition of the organic fertilizer “Bioorganik”. Table S2: Composition of the mineral fertilizer “Rosasol K”.
