# Supplementary material for: Spent Mushroom Substrate Improves Microbial Quantities and Enzymatic Activity in Soils of Different Farming Systems
Source: Microorganisms. 2024 Jul 24;12(8):1521. doi: 10.3390/microorganisms12081521 (PMC11356570; doi:10.3390/microorganisms12081521)
Supplement: Supplementary file 1 [file microorganisms-12-01521-s001.zip › microorganisms-3104758-supplementary.pdf]

Table S1. Composition of the organic fertilizer "Bioorganik".

Nutrient	Nutrient content (%)
Total N	5
P	0.1
K_2O	0.02
Ca	16
Total Mg	2
B	0.1
Cu	0.02
Fe	1.5
Mn	0.02
Zn	0.12

Table S2. Composition of the mineral fertilizer "Rosasol K".

Nutrient	Nutrient content
Total N	12 %
P	12 %
K_2O	36 %
B	100 ppm
Cu	75 ppm
Fe	260 ppm
Mn	320 ppm
Zn	230 ppm
